# Computer Simulations and Network-Based Profiling of Binding and Allosteric Interactions of SARS-CoV-2 Spike Variant Complexes and the Host Receptor: Dissecting the Mechanistic Effects of the Delta and Omicron Mutations

**DOI:** 10.3390/ijms23084376

**Published:** 2022-04-15

**Authors:** Gennady Verkhivker, Steve Agajanian, Ryan Kassab, Keerthi Krishnan

**Affiliations:** 1 Keck Center for Science and Engineering, Graduate Program in Computational and Data Sciences, Schmid College of Science and Technology, Chapman University, Orange, CA 92866, USA; agaja102@mail.chapman.edu (S.A.); rkassab@chapman.edu (R.K.); kekrishnan@chapman.edu (K.K.); 2 Department of Biomedical and Pharmaceutical Sciences, Chapman University School of Pharmacy, Irvine, CA 92618, USA

**Keywords:** SARS-CoV-2 spike protein, ACE2 host receptor, molecular dynamics, network analysis, mutational scanning, binding energetics, allosteric communications, signal transmission

## Abstract

In this study, we combine all-atom MD simulations and comprehensive mutational scanning of S-RBD complexes with the angiotensin-converting enzyme 2 (ACE2) host receptor in the native form as well as the S-RBD Delta and Omicron variants to (a) examine the differences in the dynamic signatures of the S-RBD complexes and (b) identify the critical binding hotspots and sensitivity of the mutational positions. We also examined the differences in allosteric interactions and communications in the S-RBD complexes for the Delta and Omicron variants. Through the perturbation-based scanning of the allosteric propensities of the SARS-CoV-2 S-RBD residues and dynamics-based network centrality and community analyses, we characterize the global mediating centers in the complexes and the nature of local stabilizing communities. We show that a constellation of mutational sites (G496S, Q498R, N501Y and Y505H) correspond to key binding energy hotspots and also contribute decisively to the key interfacial communities that mediate allosteric communications between S-RBD and ACE2. These Omicron mutations are responsible for both favorable local binding interactions and long-range allosteric interactions, providing key functional centers that mediate the high transmissibility of the virus. At the same time, our results show that other mutational sites could provide a “flexible shield” surrounding the stable community network, thereby allowing the Omicron virus to modulate immune evasion at different epitopes, while protecting the integrity of binding and allosteric interactions in the RBD–ACE2 complexes. This study suggests that the SARS-CoV-2 S protein may exploit the plasticity of the RBD to generate escape mutants, while engaging a small group of functional hotspots to mediate efficient local binding interactions and long-range allosteric communications with ACE2.

## 1. Introduction

The rapidly growing body of structural and functional studies has established that the mechanism of SARS-CoV-2 infection involves conformational transitions between distinct functional forms and the activation of the viral spike (S) glycoprotein trimer, which consists of an amino (N)-terminal S1 subunit and carboxyl (C)-terminal S2 subunit where S1 participates in the interactions with the host receptor and includes an N-terminal domain (NTD), the receptor-binding domain (RBD), and two structurally conserved subdomains SD1 and SD2 [1,2,3,4,5,6,7,8,9]. Conformational transitions between the closed S state with RBDs in the “down” conformation and the receptor-bound open state in which RBDs can adopt “up” conformation were characterized using biophysical experiments [10,11,12], suggesting that the mechanisms of conformational selection and receptor-induced structural adaptation can often involve allosteric stabilization and regulation. Cryo-EM and X-ray structures of the SARS-CoV-2 S complexes with different classes of potent antibodies have revealed multiple conformation-dependent epitopes, highlighting the link between the conformational plasticity and adaptability of S proteins and their capacity for eliciting specific binding and broad neutralization responses [13,14,15,16,17,18,19,20,21,22,23,24,25,26].

**Figure 1:**
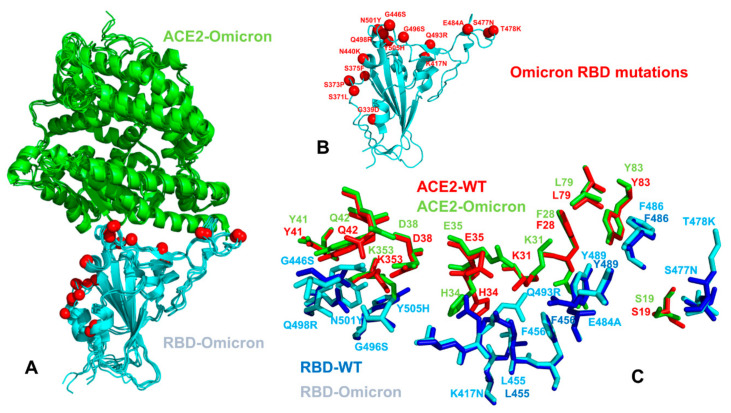
Structural organization of the SARS-CoV-2-RBD Omicron complexes with the human ACE enzyme. (**A**) Structure superposition of three structures of the SARS-CoV-2-RBD Omicron complexes with ACE2 (pdb id 7WBP, 7WBL and 7T9L). The SARS-CoV-2-RBD is shown in aqua-blue-colored ribbons, the bound ACE2 enzyme is in green ribbons and the Omicron mutational sites are highlighted in red spheres. (**B**) An overview of the SARS-CoV-2-RBD Omicron structure (pdb id 7WBP). The S-RBD is in aqua-blue-colored ribbons and sites of Omicron mutations are shown in red spheres. (**C**) A closeup of the binding interface residues in the SARS-CoV-2-RBD WT and SARS-CoV-2 Omicron RBD complexes with ACE2. The S-RBD WT interface residues are shown in navy blue sticks and the S Omicron RBD residues are in cyan sticks. The ACE2 residues from the binding interface with S-RBD WT are in red sticks and the ACE2 interfacial residues from the complex with S Omicron RBD are shown in green sticks. The binding interface residues are annotated.

The emergence of variants of concern (VOC) with an enhanced transmissibility and infectivity profile [27,28,29,30,31,32,33,34,35,36,37,38,39,40,41,42,43,44] unveiled the complex mechanisms underlying the function and dynamics of the S proteins. The recent VOC, omicron (B.1.1.529), which displays a number of mutations in the S-RBD regions, has further intensified the scientific and public interest and concerns about the role and mechanisms underlying the emergence of variants [45,46,47,48,49]. Omicron bears 37 mutations in its S protein relative to the original SARS-CoV-2 strain, with 15 mutations featured in its RBD (Figure 1). In particular, nine mutations are located within the receptor-binding motif (RBM) interacting directly with ACE2. The latest structural investigation convincingly demonstrated that mutational diversity of Omicron (B.1.1.529) can induce a widespread escape from neutralizing antibody responses [48]. According to this study, the mutations S477N, Q498R and N501Y increase ACE2 affinity by 37-fold, serving to anchor the RBD to ACE2, while allowing the RBD region freedom to develop further mutations, including those that reduce the ACE2 affinity in order to evade the neutralizing antibody response [48]. The comparison of binding affinity changes using a randomly mutated RBD screen displayed on the yeast surface for ACE2 binding showed the higher affinity for N501Y, E484K, S477N and Q498R, where the combination of Q498R and N501Y increased the binding affinity by 26-fold and adding the S477N mutation produced a 37-fold increase in the binding affinity [48]. Similar binding affinity patterns were observed in biophysical studies of the Omicron RBD interactions with ACE2 using surface plasmon resonance (SPR) experiments, which demonstrated a tighter binding in comparison to other variants [49]. Mutations present in the Omicron virus (S477N, E484K and N501Y) were preferentially selected by yeast surface display affinity maturation screen of RBD against ACE2 [50]. Recent high-profile structural biology studies of the Omicron variant of the S protein in various functional states and complexes with the ACE2 and antibodies have provided the initial rationale on which molecular features are responsible for high transmissibility, while escaping immune defense. The crystal and cryo-EM structures of the Omicron RBD–ACE2 complex and the X-ray structure of the Delta RBD–ACE2 complex identified the role of key residues for receptor recognition, showing that the mutations S477N, Q493R, Q498R and N501Y can enhance the binding affinity of RBD with ACE2, while T478K may influence ACE2 binding allosterically [51]. Several structural studies suggested that the improved electrostatic complementarity induced by the L452R and T478K substitutions accounts for a moderate increase in ACE2 affinity [52]. Another cryo-EM investigation of the S Delta variant binding noticed that L452R is not involved in direct contacts with ACE2, while a T478K substitution could induce the conformational change of the RBM loop and strengthen the interaction with the ACE2 receptor, leading to the enhanced transmissibility of the Delta variant [53]. Other structural studies found that membrane fusion for the S Delta protein is substantially faster than that of other variants, whereas the Delta mutational sites L452R and T478K are not in direct contact with ACE2 and have a smaller effect on ACE2 affinity [54]. The cryo-EM structural analysis of the Omicron variant in the unbound form and in the complex with ACE2 revealed that the interactions mediated by the mutational sites Q493R, G496S, Q498R and N501Y may restore the ACE2 binding strength that is partly compromised by other mutations [55]. SPR studies showed a measurable increase in the affinity for ACE2 relative to the S wild-type (WT) protein, suggesting that the Omicron variant may have evolved to mediate the neutralization escape via multiple mutations, while simultaneously enhancing the binding affinity with ACE2 through the acquisition of strong interactions by several energy hotspots [55]. The cryo-EM analysis discovered the compact closed and open states of the Omicron S trimers, showing that Omicron substitutions can markedly stabilize the Omicron S trimer by strengthening the allosteric interaction network between neighboring protomers and between the S1 and S2 subunits [56]. This study found that the enhanced binding affinity of the S Omicron trimer to ACE2 is comparable to that of the S Delta, but is higher than that of the S-G614, which may be due to the increased RBM–ACE2 interaction network contributed by the Q493R, and Q498R, G496S and Y505H mutant sites. The cryoEM structures of the SARS-CoV-2 Omicron S ectodomain trimer bound to the S309 and S2L20 antibodies and the X-ray crystal structure of the Omicron RBD in complex with the human ACE2 elucidated the structural basis of Omicron immune evasion and receptor engagement [57]. By examining the structural and energetic bass of Omicron binding with ACE2, this study similarly asserted that the favorable interactions formed by the mutated sites S477N, Q493R, Q496S, Q498R and N501Y to ACE2 could compensate for the loss of polar interactions caused by the mutations K417N and E484A [57]. Together, these mutations induced a net enhancing effect on the binding of the Omicron RBD to human ACE2, suggesting a structural basis for immune evasion, while preserving efficient host receptor engagement.

The structural and biochemical characterization of the Omicron S trimer and its binding to ACE2 demonstrated that ACE2 can stabilize the S trimer in the 1-RBD-up state through the formation of favorable inter-protomer RBD–RBD contacts and improved ACE2–RBD interfacial interactions, both of which contribute to the 6–9-fold increased affinity of the Omicron S protein as compared to the S-WT trimer [58]. The structural diversity of the SARS-CoV-2 Omicron S protein was elucidated using the cryo-EM structures of the Omicron and Delta variants in different functional states, showing a reduced S1 variability with immune-evasive RBD substitutions stabilizing the RBD–RBD interfaces in the 3-RBD-down structure [59]. Importantly, this pioneering study established that, while other variants favor open-states and immune evasion by mutating common antibody epitopes, the mutant-induced stabilization of the Omicron RBD-down state represents a different mechanism that promotes immune evasion by occluding highly immunogenic sites [59]. The cryo-EM structures of the Omicron S trimer solved at serological and endosomal pH were consistent with other structural studies and thermal stability assays verified that the Omicron S trimer was more stable than the WT and Delta variants [60]. The structural analysis of the key interactions at the interface with ACE2 confirmed that T478K, Q493R, G496S and Q498R could strengthen the binding of the S Omicron to ACE2, with the N501Y mutation alone improving the binding affinity by sixfold [61]. The cryo-EM study of the full-length S protein of the Omicron variant [62] analyzed the binding and antigenic properties of the Omicron S trimer by bio-layer interferometry (BLI), confirming the improved binding due to the N501Y, Q493R and Q498R mutations, as was similarly asserted in recent studies [57,58,59,60,63]. Several other recent cryo-EM structural studies supported the proposed mechanism in which the Omicron mutations can stabilize the S trimer in the 1-RBD-up open and closed state, allowing for receptor binding, while concealing the epitopes that are the targets of neutralizing antibodies [63]. According to these studies, the Omicron mutations S477N, T478K and E484A targeting the flexible region of the RBM together with K417N account for the trade-off between a moderate loss of ACE2 binding and the neutralization escape potential of the Omicron variant from the antibodies that target RBM epitopes.

Computer simulations and protein modeling have played a key role in shaping up our understanding of SARS-CoV-2 glycoproteins [64,65,66,67,68,69,70,71,72,73,74,75,76,77,78,79,80], revealing the conformational diversity and structural adaptability of the S proteins. Computational and biophysical kinetics studies of the SARS-CoV-2 S trimer interactions with ACE2 using the recent crystal structures have also provided important insights into the key determinants of the binding affinity and selectivity [71,81,82,83]. Our studies suggested that the SARS-CoV-2 spike protein can function as an allosteric regulatory engine that fluctuates between dynamically distinct functional states [84,85,86,87,88,89,90]. Structural and computational analysis analyzed the variations of interactions with ACE2 between the Omicron RBD and WT-RBDs in their binding to the ACE2 receptor [91]. MD simulations combined with the Markov state modeling of conformational states and binding free energy calculations identified four key mutations (S477N, G496S, Q498R and N501Y) for the enhanced binding of ACE2 by the Omicron RBD [91]. The strength of interactions between Omicron RBD and ACE2 was investigated using MD simulations, confirming the greater affinity of the Omicron compared to the S-WT protein [92]. Computational mutagenesis and free energy perturbation analysis showed that Omicron RBD binds to ACE2 approximately 2–3 times stronger than the native S protein, with three mutational sites T478K, Q493K and Q498R, enhancing binding affinity through more favorable electrostatic interactions [93]. Molecular dynamics (MD) simulations were combined with microscale thermophoresis to examine the binding between Omicron RBD and ACE2, showing the enhanced RBD–ACE2 interactions of the N501Y, Q493R and T478K mutational sites and revealing that the Omicron RBD exhibits a five-fold higher binding affinity to ACE2 compared to the native RBD [94]. Computer simulations of the S Omicron RBD structures and complexes with ACE2 suggested enhanced interfacial interactions induced by mutations and the increased stabilization of the binding interface with the ACE2 receptor [95,96,97]. While computational studies examined the local interactions of the Omicron RBD with ACE2, it remains unclear if this constellation of mutations could significantly perturb or alter the long-range couplings and communications between the S protein and host receptor that are important to understand the mechanisms of the increased transmissibility of this variant. Recent network-based analysis offered interesting insights into RBD–antibody interactions, showing that Omicron mutations could broadly and deeply perturb networks across different epitope classes [98]. This study suggested that network analysis may help to capture the subtle effects of distant Omicron mutations on immune escape.

In this study, we combine all-atom MD simulations, comprehensive mutational scanning and ensemble-based global network analysis of the interaction networks to quantify the local and global effects of mutations in the S-RBD complexes with ACE2. We examine and compare dynamic, energetic and networking properties for the S-RBD Delta and Omicron variants to identify the critical binding hotspots and allosteric functional centers. Complementary to this analysis, we also examine differences in allosteric interactions and communications in the S-RBD complexes for the Delta and Omicron variants. Though the perturbation-based scanning of the allosteric propensities of the SARS-CoV-2 S-RBD residues and dynamics-based network centrality and community analyses, we characterize the global mediating centers in the complexes and the nature of local stabilizing communities. Despite the remarkable structural similarities of the S-RBD complexes, small dynamic differences in the binding interfaces and mutational sites can induce measurable changes in the allosteric interaction network and determine the mechanism of allosteric communication in the Omicron complex. We show that a constellation of mutational sites (G496S, Q498R, N501Y and Y505H) correspond to key binding energy hotspots and also contribute decisively to the key interfacial communities that mediate allosteric communications between S-RBD and ACE2. These Omicron mutations are responsible for both the favorable local binding interactions and long-range allosteric interactions, providing key functional centers that mediate the high transmissibility of the virus. At the same time, our results show that other mutational sites could provide a “flexible shield” surrounding the stable community network, thereby allowing the Omicron virus to modulate immune evasion at different epitopes, while protecting the integrity of binding and allosteric interactions in the RBD–ACE2 complexes. This study suggests that the SARS-CoV-2 S protein may exploit the plasticity of the RBD to generate escape mutants that alter the response to antibody binding without compromising the binding affinity with ACE2. By leveraging the advantages afforded by network modeling and combining molecular simulations with the perturbation-based analysis of allosteric interactions, our approach can provide a useful complementary framework to support more rigorous and detailed mesoscale simulations of SARS-CoV-2 S proteins.

## 2. Results and Discussion

### 2.1. Atomistic Modeling and Simulations Reveal Distinct Conformational Flexibility Patterns of the SARS-CoV-2 S Mutant Variants

**Figure 2:**
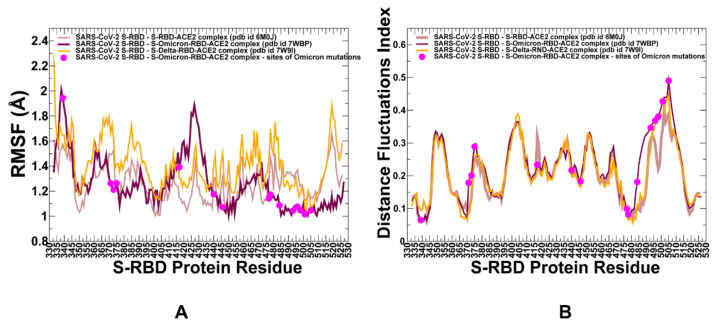
Conformational dynamics profiles obtained by averaging results from 10 independent MD simulations of the SARS-CoV-2, S-RBD complexes with ACE2 for the WT S-RBD, S Delta RBD and S-Omicron RBD. (**A**) The RMSF profiles for the RBD residues obtained from the simulations of the S-RBD WT complex with ACE2, pdb id 6M0J (in light brown lines); S Omicron RBD complex with ACE2, pdb id 7WBP (in maroon lines); and S Delta RBD complex with ACE2, pdb id 7W9I (in orange lines). The positions of the Omicron mutational sites are highlighted in magenta-colored filled circles. (**B**) The distance fluctuations stability index obtained from the simulations of the S-RBD WT complex with ACE2, pdb id 6M0J (in light brown lines); S Omicron RBD complex with ACE2, pdb id 7WBP (in maroon lines); and S Delta RBD complex with ACE2, pdb id 7W9I (in orange lines). The positions of the Omicron mutational sites are highlighted in magenta-colored filled circles.

We performed ten independent all-atom MD simulations for each of the studied protein systems: the S WT RBD–ACE2 complex, S Delta RBD–ACE2 complex and S Omicron RBD–ACE2 complex (Figure 1). This allowed for a detailed analysis of the conformational landscapes and variant-induced differences in the conformational mobility. In the Omicron RBD–ACE2 complex, a considerable number of the binding interfaces (G446, Y449, F486, Y489, G496S, Q498R, T500, N501Y and Y505H) become stabilized to make strong specific interactions with ACE2 (Figure 2A). We observed that the Omicron mutations induce the stabilization of the RBD interface, including mobile flexible RBM loops (residues 473–487). Conformational dynamics profiles were described using the root-mean-square fluctuations (RMSF) obtained from the simulations (Figure 2). The conformational mobility distribution for the Omicron RBD featured several deep local minima, corresponding to residues 374–377, the RBD core residue cluster (residues 396–403), residues 445–456, which contain β-sheet β5 (residues 451–454), and residues 496–505 that form a critical cluster of binding interface residues (Figure 2A). Specifically, a flexible motif consisting of residues 364–375, which contains the mutations S371L, S373P and S375F, shows smaller fluctuations in the Omicron complex as compared to the S Delta complex (Figure 2A). These mutated positions make non-specific interactions with F342, A435 and W436 in the hydrophobic pocket and create a fairly stable local interaction cluster that is unique for the S Omicron RBD–ACE2 complex. In general, the flexible portion of the cryptic binding site of the RBD is more stable in the S Omicron RBD complex. The portion of the RBM region (residues 446–494) that provides the contact interface with ACE2 also displayed appreciably smaller movements and became largely stabilized in the Omicron RBD complex (Figure 2A). The reduced RMSF values are especially pronounced at the flexible loop (residues 475–490) of the Omicron RBD that contains the mutational sites S477N and T478K. The thermal fluctuations of the residues 496–505 in the Omicron and Delta RBD complexes are similar and only marginally larger than in the native complex. These changes could partly reflect the greater adaptability and plasticity of the Omicron mutational sites in this region (G496S, Q498R, N501Y and Y505H) that can strengthen the binding interface contacts. The important dynamic signature of the S Omicron RBD complex is a curtailed mobility of the RBM tip that includes the functional positions S477N, T478K and E484A (Figure 2A). This can be contrasted with the dynamics profile in this region for the native S-RBD and S Delta-RBD complexes that showed a similar and more flexible behavior. This is in line with the structure-based analysis of the RBD–ACE2 complexes, showing that the Omicron RBD–ACE2 interface has better optimized interactions compared to that of the S Delta RBD [51]. Another area of dynamic differences corresponds to a flexible region 380–390 that does not contain Omicron mutations, but close to the constellation of S317L, S373P and S375F mutational sites (Figure 2A). In general, our results show the increased stabilization of the S Omicron-RBD complex as compared to the S-WT RBD complex, where not only the interfacial regions of the RBD and ACE2 proteins become more stable, but the entire complex displayed smaller fluctuations. These observations pointed to the increased stability of the S Omicron RBD. By averaging the time-dependent evolution of specific interactions over independent MD simulations, we detected the stability and high occupancy of several important interfacial hydrogen bonds and specific interactions formed by N477, R493, S496, R498 including N477–S19 (34% occupancy), N477–Q24 (32%) Y453–H34 (78%) R493–H34 (62%), R493–E35 (54%), R493–K31 (59%), Y501–K355 (78%), Y501–D38 (82%), Y501–K353 (85%), K417–D30 (63%), R493–E35 (58%), R498–D38 (61%), R498–Q42 (53%), R498–Y41 (73%) and H505–E37 (68%). Notably, Y501 and H505 together with the R498 site form a stable cluster that restricts the movements of the Omicron positions and can stabilize the binding interface. These stable interactions result in the increased rigidification of the negatively charged ACE2 residues D30, E35, E37 and D38 and highly positive binding interfacial sites on the S Omicron RBD, which provide a driving force of the increased binding energetics for the Omicron variant.

The conformational ensembles of the S-RBD complexes with ACE2 were also subjected to a distance fluctuations coupling analysis based on the dynamic residue correlations (Figure 2B). The residue-based distance fluctuation communication indexes measure the energy cost of the dynamic residue deformations and could serve as a robust metric for the assessment of structural stability [99,100] and the allosteric propensities of protein residues [87,88,89]. In this model, dynamically correlated residues, whose effective distances fluctuate with low or moderate intensity, are expected to communicate with higher efficiency than the residues that experience large fluctuations. Notably, structurally stable and densely interconnected residues as well as moderately flexible residues that serve as a source or sink of allosteric signals could have a high value of these indexes. This model allows for the identification of regions with high structural stability and allosteric potential. In all complexes, the core regions of the RBD experienced small thermal fluctuations and corresponded to the distribution peaks, thus signaling structural stability of these regions (Figure 2B). The dominant peaks of the distance fluctuation profiles correspond to functionally important stability centers (F400, I402, Y505, Y508, F490, Y489 and A475) in all complexes. Although the shapes of the dynamic profiles in the S-RBD complexes with the ACE2 are similar, there are relevant differences that are reflected in binding-induced flexibility changes (Figure 2B). Interestingly, we found increased values for the distance fluctuation stability index of the Q493R, G496S and Q498R positions in the S Omicron RBD complex with ACE2. Hence, Omicron mutations in these positions induce the increased stabilization of these residues in the ACE2 complex compared to the native S-RBD and S Delta RBD complexes (Figure 2B). These results also suggested that the key mutated residues Q493R, G496S and Q498R contribute to the increased stability of the complex and also acquire a stronger allosteric potential to mediate long-range allosteric interactions between S-RBD and ACE2.

**Figure 3:**
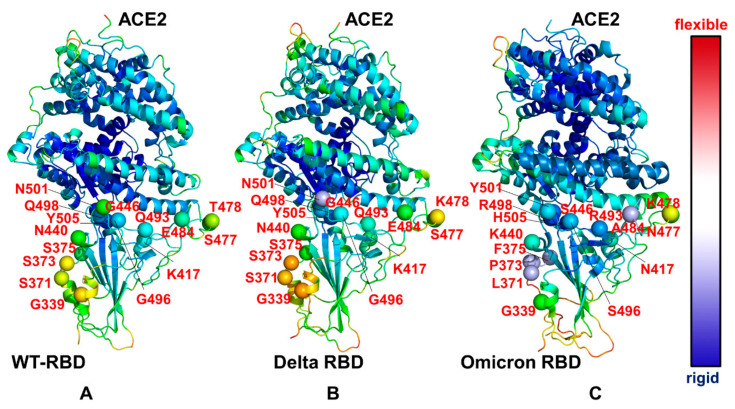
Structural maps of the conformational profiles obtained from the simulations of the SARS-CoV-2 S-RBD variant complexes. Conformational mobility map obtained from the simulations of the S-RBD WT complex with ACE2, pdb id 6M0J (**A**); S Delta RBD complex with ACE2, pdb id 7W9I (**B**); and S Omicron RBD complex with ACE2, pdb id 7WBP (**C**). The structures are shown in ribbons with the rigidity–flexibility sliding scale colored from blue (most rigid) to red (most flexible). The positions of sites targeted by Omicron mutations are shown in spheres colored according to their mobility level. The positions are highlighted and fully annotated on all panels. Note the progressive stabilization of the binding interface regions and mutational sites in the S Omicron RBD complex.

The structural maps of the conformational mobility profiles illustrated the overall progressive stabilization of the RBD–ACE2 complex from the S-WT RBD (Figure 3A) to S Delta RBD (Figure 3B) and S Omicron RBD (Figure 3C). To highlight the dynamic changes induced by the mutations, we mapped all positions of the Omicron mutations on the S RBD complexes (Figure 3). It is apparent that that the residues G339, S371, S373, S375, N440 and G446 of the native S-RBD are located in the more flexible regions and retain an appreciable degree of plasticity in the complex with ACE2 (Figure 3A). At the same time, the key interfacial centers, including Q498, N501 and Y505, remain stable in the native complex, forming a dense interacting cluster with ACE2 (Figure 3A). The RBD residues K417, Q493 and G496 showed moderate mobility, while the RBM positions E484, S477 and T478 displayed a considerable flexibility. In the S Delta RBD–ACE2 complex, the overall distribution of conformational mobility was similar to that of the native complex, showing a stabilization of the Q493, Q498, N501 and Y505 residues and a moderate flexibility of L452R and T478K (Figure 3B).

A more dramatic change in the conformational dynamics map can be seen for the S Omicron RBD complex with ACE2 where the majority of mutational sites undergo a significant stabilization and experience smaller thermal fluctuations (Figure 3C). Importantly, the mutational sites in the RBD interfacial region (S446, A484, R493, S496, R498, Y501 and H505) become more stable in the S Omicron RBD complex as compared to the S-WT RBD and S Delta RBD complexes with ACE2. The results of the simulations demonstrate the increased stabilization of the binding interface for the S Omicron RBD–ACE2 complex and the enhanced number of stable intermolecular interactions, which is consistent with other studies showing the weaker interfacial interactions in S Delta compared to S Omicron [95,96,97]. The differences in the thermal fluctuations of the ACE2 residues in the complexes are rather moderate, showing minor motions of the N-terminal helix of ACE2 closer to the RBD in the Omicron complex (Figure 3). Of interest, we noted the increased rigidity of the S-RBD core residues and also ACE2 regions that are distant from the inter-molecular binding interface (Figure 3C). This indicated that Omicron mutations, by forming stronger interfacial contacts, may also induce restricted dynamic variations and reduce conformational diversity over a longer range in the peripheral RBD regions and remote ACE2 residues. These observations are consistent with our hypothesis that key Omicron mutations, including Q493R, G496S and Q498R, display a stronger allosteric potential in the Omicron complex and therefore may allosterically modulate the stabilization of the RBD and ACE2 regions.

### 2.2. Ensemble-Based Mutational Sensitivity Analysis Identifies Key Structural Stability and Binding Affinity Hotspots in the SARS-CoV-2 RBD Complexes with ACE2

**Figure 4:**
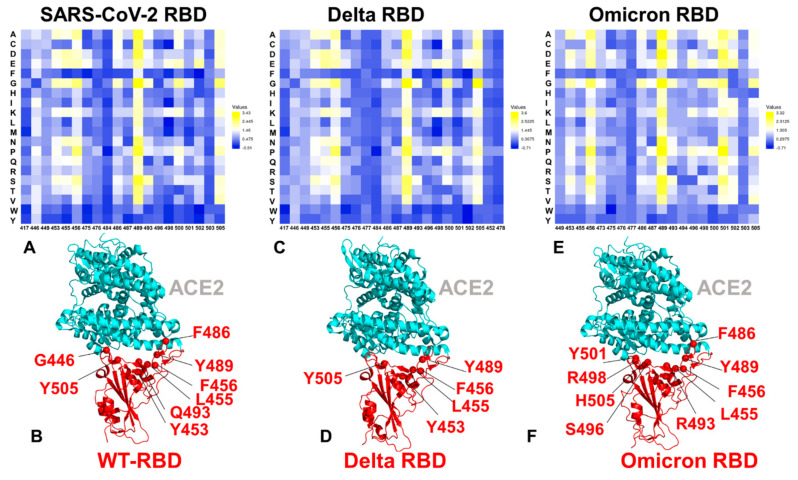
Ensemble-based mutational scanning of protein stability and binding for the SARS-CoV-2 S-RBD complexes with ACE2. (**A**) The mutational scanning heatmap for the S-RBD WT complex with ACE2. The binding energy hotspots correspond to residues with high mutational sensitivity. (**B**) Structural map of the binding energy hotspots for the S-RBD WT complex. S-RBD is in red ribbons and ACE2 is in cyan ribbons. The energetic hotspots are shown in red spheres and annotated. (**C**) The mutational scanning heatmap for the S Delta RBD complex with ACE2. (**D**) Structural map of the binding energy hotspots for the S-RBD WT complex. The energetic hotspots are shown in red spheres and annotated. (**E**) The mutational scanning heatmap for the S Omicron RBD complex with ACE2.; (**F**) Structural map of the binding energy hotspots for the S-RBD WT complex. The energetic hotspots are shown in red spheres and annotated. Mutational scanning was performed for the S-RBD interfacial residues calculated using the BeAtMuSiC approach [101,102,103]. The heatmaps show the computed binding free energy changes for 20 single mutations on the sites of variants. The squares on the heatmap are colored using a 3-colored scale blue–white–yellow, with yellow indicating the largest unfavorable effect on stability. The standard errors of the mean for binding free energy changes were based on a different number of selected samples from a given trajectory (500, 1000 and 2000 samples) are within 0.12–0.22 kcal/mol.

By employing the conformational ensembles of the S-RBD WT, Delta and Omicron variant complexes with ACE2, we performed the mutational scanning of the interfacial RBD residues and computed the binding free energy changes. In silico mutational scanning was performed using the BeAtMuSiC approach [101,102,103]. This approach allows for accurate predictions of the effect of mutations on both the strength of the binding interactions and the stability of the complex using statistical potentials and neural networks. The adopted BeAtMuSiC approach in our study was further enhanced through the ensemble-based averaging of binding energy computations. The binding free energy ΔΔG changes were computed by averaging the results of computations over 1000 samples obtained from simulation trajectories. To provide a comparison between the computational and experimental data, we constructed mutational heatmaps for the SARS-CoV-2 S-RBD binding interface residues (Figure 4).

Although structural and dynamic similarities yield fairly similar energetic heatmaps, there are a number of notable differences that provide insight into the binding mechanisms of the Omicron variant. Consistent with deep mutagenesis experiments [104,105,106,107], the strongest stability and binding energy hotspots correspond to the Y453, F456, Y489 and Y505 residues (Figure 4A,B). Notably, essentially all modifications in these positions resulted in a considerable loss of S-RBD stability and binding affinity with ACE2. These observations are also consistent with the SARS-CoV-2 binding selectivity influenced by the virus-binding hotspot K31 and H34 in the middle of the interface forming an extensive interaction network with the Y453, L455, F456 and Q493 residues. The common energetic hotspots Y453, Y489 and Y505 found in mutational scanning also emerged as critical stability and binding hotspots in deep mutagenesis studies, which demonstrated that RBD stability contributes to ACE2-binding affinity [104,105,106,107]. In general, mutations that improve stability and rigidity often accompany increases in binding affinity. The S-RBD sites Q493, Q498 and N501 in the S-WT RBD complex with ACE2 showed that a number of mutational changes can improve the interactions. Mutational scanning revealed that the mutations Q493R, Q498R and N501Y in the native complex could be moderately stabilizing, yielding ΔΔG = −0.12 kcal/mol, ΔΔG = −0.22 kcal/mol and ΔΔG = −0.28 kcal/mol, respectively (Figure 4A,B).

The mutational scanning heatmap for the S-RBD Delta RBD binding with ACE2 revealed a narrower spectrum of binding energy hotspots featuring the Y453, L455, Y489 and Y505 residues (Figure 4C,D). It is evident that mutations in the Q493 and Q498 positions incur only moderate destabilization changes, while modifications in the N501 site often result in an improved binding affinity with ACE2 (Figure 4C,D). Although Delta mutational sites are not in direct contact with the ACE2 receptor, we included these sites in the mutational scanning heatmap to obtain a quantitative estimate of their contributions to stability and binding (Figure 4C). The results show that L452R is only moderately favorable with mutations in this position inducing small destabilization changes, while the exposed T478K site contributes little to the RBD binding with mutational changes incurring negligible changes to the binding free energies (Figure 4C). Several structural studies suggested that the Delta variant T478K extends its positively charged sidechain towards an electronegative region on ACE2 and could improve the electrostatic complementarity accounting for the very moderate increase in ACE2 affinity [52]. Another cryo-EM investigation of the S Delta variant binding noticed that L452R is not involved in direct contacts with ACE2, while the T478K substitution was attributed to the small conformational change of the RBM loop that could strengthen the interaction with the ACE2 receptor [53]. Other structural studies found that the Delta mutational sites L452R and T478K are not in direct contact with ACE2, showing a small effect on the binding affinity in the biophysical analysis of both the full-length S Delta trimer and the S-RBD fragment alone. [53]. According to our results, the RBD-Delta mutational sites L452R and T478K have a very minor effect on the binding affinity, while the greater contribution of the neighboring Y453, L455 and F456 residues to protein stability and RBD–ACE2 interactions (Figure 4C,D) may explain the moderately improved binding of the Delta variant. Based on these results, we argue that the functional role of the mutational sites L452R and T478K may be primarily associated with the immune evasion from antibodies, rather than the appreciable contribution to the improved binding with ACE2.

The mutational heatmap for the Omicron variant RBD interfacial residues showed a number of distinct characteristics (Figure 4E,F). First, we observed a much broader distribution of the energetic hotspots, where mutations in various positions induce destabilizing changes. These sensitive RBD residues include Y453, F456, F486, N487, Y489, Q493R, G496S, Q498R, T500, N501Y and Y505H (Figure 4E,F). Hence, S-RBD interactions for the Omicron variant are significantly better optimized for binding with ACE2 than the wild-type virus. The results showed that the Omicron variant preserves key stability centers Y453, F456 and Y489 while acquiring the additional binding affinity primarily through the N501Y mutated site. This is consistent with the experimental data showing that the N501Y mutation alone can improve the binding affinity by sixfold [108]. The non-canonical π–π stacking interaction between Y501 and the ACE2 residue Y41 and additional interactions with the ACE2-binding hotspot K353 provides a considerable stabilization and was previously observed in the cryo-EM structures of the SARS-CoV-2 spike N501Y mutant in complex with ACE2 [109]. According to our simulations and energetic analysis, Y501 is closer to the Y41 and K353 residues of ACE2 than N501 in the S-WT RBD, leading to stronger binding interactions. Instructively, the N501 position does not belong to strong binding energy hotspots in the native S-RBD and S Delta RBD complexes, while the N501Y site is one of the strongest energetic hotspots in the S Omicron RBD complex, confirming the decisive role of the Y501 interaction with ACE2 in enhancing the binding strength. Indeed, all modifications in the Y501 position of the Omicron complex produced consistently significant destabilization changes (Figure 4E,F). Moreover, the largest destabilizing mutations in this position are Y501P (ΔΔG = 3.12 kcal/mol), Y501S (ΔΔG = 2.34 kcal/mol), Y501N (ΔΔG = 2.25 kcal/mol), Y501A (ΔΔG = 2.41 kcal/mol) and Y501E (ΔΔG = 2.24 kcal/mol) (Figure 4E). These results are consistent with deep mutagenesis studies and suggest that N501Y is optimally positioned for interactions with ACE2 [104,105,106,107]. In the Omicron RBD–ACE2 complex, Q493R forms new salt bridges with E35, Q498R makes new contacts with D38 and Q42, and G496S and Y505H form new hydrogen bonds with K353 (Figure 4E,F). As a result, a consistent destabilization pattern of binding free energy changes was seen for the R493, S496, and R498 positions of the Omicron RBD. Although the destabilization changes in these positions are less dramatic than for the N501Y position, we found that the reverse mutations R493Q, S496G and R498Q can result in ΔΔG = 0.7–1.2 kcal/mol (Figure 4E,F). The mutational scanning heatmap for the S Omicron RBD residues showed that the largest and consistent destabilization changes are observed for the Y489 and Y501 residues (Figure 4E). Strikingly, all modifications in these positions result in a significant loss of stability and binding affinity. These results also confirmed that Y489 is a critical stability hotspot shared by the S-WT RBD, Delta variant and Omicron variant. Mutations in this position result in a considerable loss of stability and binding interactions with ACE2. Another group of the interfacial RBD residues that make critical contributions to the protein stability are the Y453, L455 and F456 positions. Mutations in these positions showed a consistent destabilization pattern for both the Delta and Omicron variants. Notably, these hydrophobic RBD sites are not targeted by circulating variants, indicating their importance for the folding and stability of the RBD protein that needs to be preserved to enable spike activity and binding with the host receptor.

Our results indicate that the Y501 position is the most critical binding affinity hotspot in the S Omicron RBD complex with ACE2. Moreover, a tight cluster of binding affinity hotspots in this region, formed by the Y501, R498, S496 and H505 residues, makes the dominant contribution to the binding affinity (Figure 4E). The structural mapping of the binding energy hotspots in the Omicron RBD-ACE2 complex showed that the key anchoring cluster of the energetic centers is formed by the S496, R498, Y501 and H505 residues (Figure 4F). These binding energy hotspots are packed together and collectively form a dense network of the hydrophobic and polar interactions with ACE2. Notably, the binding hotspots are also formed across the ACE2 interface where Q493R and F486 provide additional stabilization. According to our results, mutations in the S477N position produce very minor destabilizing changes, while the T478K and E484A sites contribute insignificantly to the binding interface, thereby allowing for virus flexibility to produce mutations that evade neutralizing antibodies without compromising the binding affinity with ACE2. The results of mutational scanning also suggested that the enhanced binding affinity of the S Omicron RBD to ACE2, compared to the S-WT RBD complex, may be determined by significantly strengthened inter-molecular electrostatic interactions, where the Omicron mutations induce an improved contact between the positively charged residues in RBDs and the negatively charged residues in ACE2.

### 2.3. Ensemble-Based Modeling of the Residue Interaction Networks and Community Analysis in the S-RBD Complexes Reveal the Stabilization Mechanism of in the Omicron Variant

Network-centric models of protein structure and dynamics can allow for a more quantitative analysis of allosteric changes, the identification of regulatory control centers and the mapping of allosteric communication pathways. In this paper, we supported dynamics and binding energetic analysis with the modeling of the dynamic residue interaction networks that allows for a quantitative analysis of allosteric changes, the characterization of the long-range interactions and the identification of the regulatory allosteric centers that mediate allosteric communications between the S-RBD and ACE2 proteins. The residue interaction networks in the SARS-CoV-2 S-RBD structures were built using a graph-based representation of protein structures [110,111], in which residue nodes are interconnected through dynamic correlations [112]. Our previous network modeling studies of SARS-CoV-2 S proteins showed that this approach can capture allosteric interactions and communications in spike proteins [85,86,87,88,89,90]. Using the dynamic network model of residue interactions, we computed the ensemble-averaged distributions of the betweenness centrality based on average shortest path length (ASPL) change upon removing individual nodes as was initially proposed in our previous study [90]. Using these network parameters as proxy for the systematic mutational scanning of the S-RBD residues, we probed their allosteric propensities and identified the mediating centers of the allosteric interactions in the S-RBD complexes. By introducing mutational changes in the S-RBD positions, we re-evaluated dynamic inter-residue couplings and compute mutation-induced changes in the ASPL parameter. These changes were then averaged over all substitutions in a given residue node [90]. In this manner, we characterized the average mutational effect of each node on changes in the network connectivity and allosteric communications.

**Figure 5:**
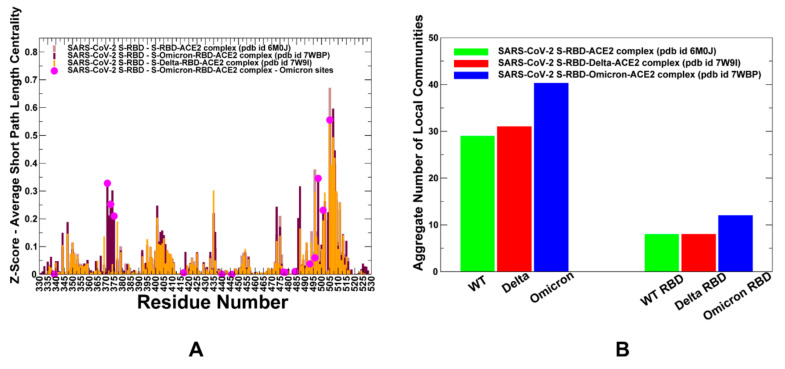
The network-based residue centrality profiles and community analysis for the S-RBD variant complexes with ACE2. (**A**) The residue-based Z-score centrality profile estimates the average mutation-induced changes in the ASPL parameter. The centrality profiles of the S-RBD residues are shown for the S-RBD WT–ACE2 complex (brown filled bars), S Omicron RBD–ACE2 complex (maroon filled bars) and S Delta RBD–ACE2 complex (orange filled bars). The positions of Omicron mutational sites are shown as magenta filled circles. (**B**) The distributions of the total number of local interacting communities in the complex (including S-RBD and ACE2 molecules) are shown on the left side of the panel and the number of local communities formed by S-RBD residues only is shown on the right side of the panel. The number of communities represents the averages obtained from the equilibrium simulation ensembles of conformations. The distribution is shown for the S-RBD WT (in green bars), S Delta RBD (in red bars) and S Omicron RBD (in blue bars).

The central questions addressed in the allosteric mutational profiling are the relationship between allosteric regulatory centers and binding free energy hotspots, as well as the linkage between mutational escape mechanisms and allosteric interactions. Through this network-based mutational scanning of allostery and by identifying residues where mutations induce a significant change in the ASPL metric (Figure 5), we located the mediating sites of long-range allosteric communication that determine the efficiency and robustness of signal transmission in the protein. The comparison of the network centrality profiles for the native S-RBD complex with ACE2 and the Delta and Omicron RBD complexes revealed distinct residue clusters that are characterized by a significant increase in mutational sensitivity (Figure 5). The mutations of these positions on average lead to an increased path length and, therefore, are referred to sites that may be important for long-range allosteric communications in the complexes. First, as may be expected from the structural and dynamic similarities, the profiles showed a similar overall shape and the presence of distinct peaks for the S-RBD core residues to sites that may be important for the efficiency of signal transmission in the complexes (Figure 5A). Among the common peaks shared by all three complexes are the S-RBD core clusters 400-VIRGDE-406 and 433-VIAW-436 (Figure 5A). A smaller, but noticeable, common peak cluster is formed by the L452, Y453, L455 and F456 residues that are anchored around the structural stability center Y453 (Figure 5A). Another common feature of the network organization is the broad peak that extends from the β4 sheet of the central RBD core (residues 507–515) to the key interfacial RBD cluster with ACE2 that includes the functional residues N501 and Y505. The comparison of the network profiles indicated a close similarity between the native S-RBD complex with ACE2 and the S Delta RBD complex (Figure 5A).

Of particular notice is the small contribution of the S Delta mutational sites L452R and T478K to the allosteric communication. We found that the efficiency of signaling between S-RBD and ACE2 is tolerant to mutations in these positions. The Omicron mutations induced critical changes in the mutational sensitivity of network centrality, revealing a broader spectrum of functional sites that could mediate signal transmission. Indeed, we observed a sharp increase in the ASPL parameter for the RBD core residues 371–376 that included the L371, P373 and F375 mutated Omicron positions (Figure 5A). In addition, larger peaks were seen for the S-RBD core positions V401 and Y473 as well as for the RBM residues F486 and N487. Moreover, a new peak was observed for the mutated Q498R position and strong peaks were also seen for the N501Y and Y505H Omicron sites. Together, these findings suggested that the key functional cluster of binding energy hotspots and allosteric communication centers is anchored by the Q498R, N501Y and Y505H residues (Figure 5A).

These functional hotspots collectively form a dense network of interconnected sites that could mediate the binding affinity and allosteric interactions in the S-RBD complex with ACE2. Mutations in these key positions can result not only in the large destabilization of the binding interactions, but also in the reduction in network efficiency by altering long-range communication routes in the SARS-CoV-2 S complexes. This analysis suggests that the structural stability centers in the S-RBD core and more dynamic mutated residues in the RBM loops can form a network of allosteric hotspots in the S Omicron complex that cooperate to propagate allosteric signals across the ACE2 binding interface. Collectively, the observed sensitivity of the short paths to sites of Omicron mutations indicated a potentially broader ensemble of communication routes that connects S Omicron RBD with the ACE2 receptor, which may be related to the higher transmissibility of the Omicron virus. Using community decomposition, the residue interaction networks were divided into local interaction modules in which residues are densely interconnected. A community-based model of allosteric communications is based on the notion that groups of residues that form local interacting communities are correlated and switch their conformational states cooperatively. In this model, allosteric communications can be transmitted through a chain of stable local modules connected via inter-community bridges. Using conformational ensembles, we computed the average number of local communities in the S-RBD complexes for the S-WT RBD complex as well as S Delta and S Omicron RBD complexes (Figure 5B). In terms of network, the greater number of stable communities is strongly linked with the overall stability of the complex and strength of the binding interfaces. We observed that the total number of communities (including both S-RBD and ACE2 molecules) is greater for the S Omicron complex, while this value is similar for the S-WT RBD and S Delta RBD complexes (Figure 5B). According to this analysis, Omicron mutations could induce the increased stabilization of the S-RBD core and also strengthen connectivity and allosteric communication across the binding interface.

### 2.4. Allosteric Communications in the S-RBD Complexes: Omicron Mutations Mediate the Increased Stability and Enhanced Allosteric Couplings

**Figure 6:**
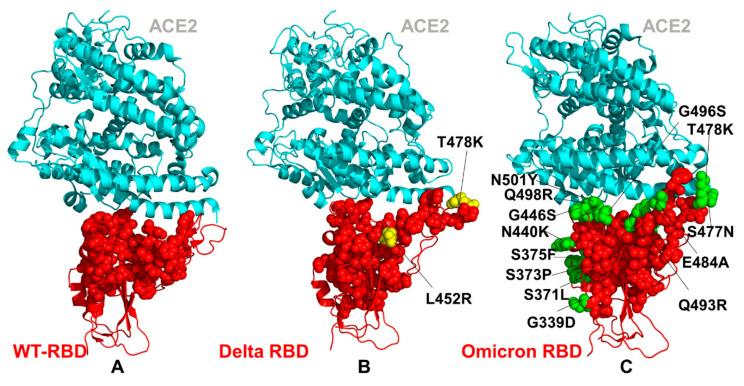
The network-based allosteric communication analysis for the S-RBD variant complexes with ACE2. The structural maps of the high edge betweenness links for the S-RBD residues are shown for the S-RBD WT–ACE2 complex (**A**), S Omicron RBD–ACE2 complex (**B**) and S Delta RBD–ACE2 complex (**C**). The S-RBD residues that participate in the high edge betweenness links are shown in spheres. The positions of the Delta mutational sites L452R and T478K on panel B and the Omicron mutational sites on panel C are shown in yellow spheres and annotated.

We leveraged the results of community decomposition and used edge betweenness (or edge centrality) in the global interaction network as a proxy for the modeling and analysis of allosteric communication pathways. This parameter is defined as the ratio of all the shortest paths passing through a particular edge to the total number of shortest paths in the network. Through the structural mapping of the high centrality edges in the most probable pathways of the ensembles, we obtained a global map of major communication routes in the interaction network of the SARS-CoV-2 S-RBD complexes (Figure 6). The topography of these maps showed the distribution and density of local communities and major communication routes. In the S-WT RBD complex, the key intermolecular community K353(ACE2)–Y41(ACE2)–Q498(RBD)–N501(RBD) is formed by Y41, K353 of ACE2 interacting with Q498 and N501 of the S-RBD. Other smaller interfacial communities involved K353(ACE2)–D38(ACE2)–Q498(RBD) and K353(ACE2)–N501(RBD)–Y505(RBD).

The structural maps highlighted the integrated role of the Q498, N501 and Y505 positions in the largest interfacial community (Figure 6A). We observed a similar number of local S-RBD communities in the S Delta RBD core and only a single interfacial community K353(ACE2)–Y41(ACE2)–Q498(RBD)–N501(RBD), which is also present in the S-WT RBD complex (Figure 5B). The structural map of allosteric communications in the S Delta RBD complex highlighted conserved local interacting modules in the RBD molecule, including F341–I434–V511, F515–L387–V382, W353–F400–Y423–I410, F464–F429–S514, R509–D442–S438, Y451–V401–D422, F342–F374–W436 and W436–R347–R509 (Figure 6B). Interestingly, for both the S-WT RBD and S Delta RBD complexes, we detected a relatively narrow communication path connecting the RBD core through L452 to a group of the stable residues F490, L492, Y489, Q493 and I472 in the RBM region (Figure 6A,B). Using this route, a communication signal from the RBD core could be transmitted to the Q493 interfacial position that is in direct contact with ACE2. According to the network analysis, F490, L492 and Y489 are stable hydrophobic sites that are firmly positioned at the binding interface and could provide stabilization anchors for signal transmission. This communication route is preserved and becomes broader in the S Delta RBD–ACE2 complex (Figure 6B). By highlighting the position of the Delta mutational sites L452R and T478K, we noticed that R452 bridges the tightly packed community cluster in the S-RBD core with more dynamic interacting modules in the RBM region (Figure 6B). The network analysis showed that the L452 and L452R positions can bridge the S-RBD core residues V350 and Y351 and local communities anchored by these sites with the hydrophobic residues Y453, L455, F490 and L492 from the key interfacial region with ACE2 (Figure 6A,B). Interestingly, L452 in the WT complex allows for stronger couplings with the hydrophobic residues along this path and enhances the allosteric communications between distant RBD regions. Accordingly, our findings indicated that the L452R and T478K mutations in the S Delta RBD complex are unlikely to have a significant effect on either local binding interactions or long-range allosteric couplings. As L442R and T478K also appeared to only moderately contribute to the binding affinity, we argue that the functional role of these mutations in modulating binding and allostery is minor. Experimental studies have shown that L452R reduces the protein reactivity with viral neutralizing antibodies and sera from convalescent patients can reduce the neutralizing activity of many RBD-specific monoclonal antibodies [113,114,115]. Our analysis suggests that the primary functional role of the L452R and T478K mutations in the Delta variant may arise not from their effect on stability, binding or allostery, but can be determined by a broad immune escape potential. Indeed, it was asserted that the strong association of the L452R mutation with immune escape could result in a stronger cell attachment of the virus, with both factors increasing viral transmissibility, infectivity and pathogenicity [113].

The community decomposition in the S Omicron RBD complex revealed a denser intramolecular network and a markedly increased number of the RBD–ACE2 interfacial communities (Figure 5B), which included Q24–Y83–N487(RBD), Y489(RBD)–K31(ACE2)–F456(RBD), F456(RBD)–K417(RBD)–D30(ACE2), N330(ACE2)–D355(ACE2)–T500(RBD), K353(ACE2)–S496(RBD)–Y501(RBD), D38(ACE2)–Y449(RB)–R498(RBD), Y41(ACE2)–K353(RBD)–Y501(RBD), K353(ACE2)–Y501(RBD)–H505(RBD) and Y41(ACE2)–Y501(RBD)–R498R(RBD). This analysis showed that the N501Y residue participates in key interfacial communities, thus enhancing the stability of the binding interface and strengthening the efficiency of the global interaction network. In addition, Q498R and G496S contributed to the newly formed interfacial clusters. These results also provide a broader interpretation of the binding contributions of these energetic hotspots as these sites enhance both the local interactions and global connectivity of the allosteric network. A significantly different and denser communication map was obtained for the S Omicron RBD–ACE2 complex (Figure 6C). It appeared that the “busiest” communication links with the highest edge centrality involve the Y505H, N501Y and Q498R sites that most efficiently link the S-RBD core with ACE2. The increased density of the communication map for the most probable routes reflects the increased heterogeneity of the interaction network for the Omicron RBD–ACE2 complex. We observed the increased communication role of the Th anti-parallel β-sheets β5 and β6 (residues 451–454 and 491–495) that connect the central core with the more flexible RBM region. The network of short paths allows for rapid signaling across the major interfacial centers N501/Y505/Q498, Q493, Y489 and F486 (Figure 6C). Hence, the results suggest that Omicron mutations could not only lead to an improved binding affinity with ACE2, but also expand the allosteric interaction network and allow for robust signal transmission via a broad ensemble of communication routes. Together, these findings argue that the enhanced Omicron infectivity and virus transmissibility may arise from the cumulative effect of binding interactions and the more efficient and resilient interaction network formed in the S Omicron RBD–ACE2 complex.

## 3. Materials and Methods

### 3.1. Structure Preparation and Analysis

All structures (Table 1) were obtained from the Protein Data Bank [116,117]. During the structure preparation stage, protein residues in the crystal structures were inspected for missing residues and protons. Hydrogen atoms and missing residues were initially added and assigned according to the WHATIF program web interface [118,119]. The structures were further pre-processed through the Protein Preparation Wizard (Schrödinger, LLC, New York, NY, USA), which included the check of bond order, assignment and adjustment of ionization states, formation of disulphide bonds, removal of crystallographic water molecules and co-factors, capping of the termini, assignment of partial charges and addition of possible missing atoms and side chains that were not assigned in the initial processing with the WHATIF program. The missing loops in the studied cryo-EM structures of the SARS-CoV-2 S protein were reconstructed and optimized using the template-based loop prediction approaches ModLoop [120] and ArchPRED server [121] and further confirmed using the FALC (Fragment Assembly and Loop Closure) program [122]. The side chain rotamers were refined and optimized using the SCWRL4 tool [123]. The protein structures were then optimized using atomic-level energy minimization with a composite physics and knowledge-based force field as implemented in the 3Drefine method [124]. The atomistic structures from simulation trajectories were further elaborated by adding *N*-acetyl glycosamine (NAG) glycan residues and optimized.

### 3.2. Molecular Dynamics Simulations

A total of 10 independent all-atom MD simulations were performed for each of the studied protein systems: the WT S-RBD–ACE2 complex, S Delta RBD–ACE2 complex and S Omicron RBD–ACE2 complex. MD simulations were conducted using an N, P, T ensemble in an explicit solvent with the NAMD 2.13 package [125] and CHARMM36 force field [126]. The long-range non-bonded van der Waals interactions were computed using an atom-based cutoff of 12 Å, switching the van der Waals potential beginning at 10 Å. Long-range electrostatic interactions were calculated using the particle mesh Ewald method [127] with a real space cut-off of 1.0 nm and a fourth order (cubic) interpolation. The SHAKE method was used to constrain all bonds associated with hydrogen atoms. Simulations were run using a leap-frog integrator with a 2 fs integration time step. Energy minimization after the addition of solvent and ions was conducted using the steepest descent method for 100,000 steps. All atoms of the complex were first restrained at their crystal structure positions with a force constant of 10 Kcal mol^−1^ × Å^−2^. Equilibration was performed in steps by gradually increasing the system temperature in steps of 20 K, starting from 10 K until 310 K, and, at each step, 1 ns equilibration was performed keeping a restraint of 10 Kcal × mol^−1^ Å^−2^ on the protein C_α_ atoms. After the restrains on the protein atoms were removed, the system was equilibrated for an additional 10 ns. An NPT production simulation was run on the equilibrated structures for 500 ns, keeping the temperature at 310 K and constant pressure (1 atm). In simulations, the Nose–Hoover thermostat [128] and isotropic Martyna–Tobias–Klein barostat [129] were used to maintain the temperature at 310 K and pressure at 1 atm.

### 3.3. Distance Fluctuations Stability and Communication Analysis

We employed distance fluctuation analysis of the simulation trajectories [99,100] to compute residue-based stability profiles. The fluctuations of the mean distance between a given residue and all other residues in the ensemble were converted into distance fluctuation stability indexes that measure the energy cost of the residue deformation during the simulations. The high values of distance fluctuation indexes are associated with stable residues that display small fluctuations in their distances to all other residues, while small values of this stability parameter would point to more flexible sites that experience large deviations of their inter-residue distances. The distance between residues used in the fluctuation metric was computed as the mean distance between each atom belonging to a given amino acid and all the atoms belonging to the remaining protein residues:

(1) $$k_{i}=\frac{3k_{B}T}{\langle {\left(d_{i}-\langle d_{i}\rangle \right)}^{2}\rangle }$$

(2) $$d_{i}={\langle d_{ij}\rangle }_{j*}$$

where $d_{ij}$ is the instantaneous distance between residue i and residue j; $k_{B}$ is the Boltzmann constant; and T = 300 K. 〈 〉 denotes an average taken over the MD simulation trajectory and $d_{i}={\langle d_{ij}\rangle }_{j*}$ is the average distance from atoms in the residue i to all other atoms in the residue j of the protein (the sum over $j_{*}$ indicates the exclusion of the atoms that belong to the residue *i*). The interactions between the C_α_ atom of residue *i* and the C_α_ atom of the neighboring residues *i* − 1and *i* + 1 were excluded from the calculation since the corresponding distances were constant. The inverse of these fluctuations yielded an effective force constant *k_i_* that described the ease of moving an atom with respect to the protein structure.

### 3.4. Mutational Scanning and Sensitivity Analysis

We conducted the mutational scanning analysis of the binding epitope residues for the SARS-CoV-2 S protein complexes. Each binding epitope residue was systematically mutated using all substitutions and the corresponding protein stability changes were computed. The BeAtMuSiC approach [101,102,103] was employed, which is based on statistical potentials describing the pairwise inter-residue distances, backbone torsion angles and solvent accessibilities, and considers the effect of the mutation on the strength of the interactions at the interface and on the overall stability of the complex. The binding free energy of protein–protein complex can be expressed as the difference in the folding free energy of the complex and folding free energies of the two protein binding partners:

(3) $$\Delta G_{bind}=G^{com}-G^{A}-G^{B}$$

The change of the binding energy due to a mutation was calculated, then, as follows:

(4) $$\Delta \Delta G_{bind}=\Delta G_{bind}^{mut}-\Delta G_{bind}^{wt}{{}_{}}^{}$$

We leveraged rapid calculations based on statistical potentials to compute the ensemble-averaged binding free energy changes using equilibrium samples from simulation trajectories. The binding free energy changes were computed by averaging the results over 1000 equilibrium samples for each of the studied systems.

### 3.5. Dynamic Network Analysis

A graph-based representation of the protein structures [110,111] was used to represent residues as network nodes and the inter-residue edges to describe non-covalent residue interactions. The network edges that define residue connectivity were based on non-covalent interactions between residue side-chains that define the interaction strength $I_{ij}$, according to the following expression used in the original studies [110,111]:

(5) $$I_{ij}=\frac{n_{ij}}{\sqrt{\left(N_{i}\times N_{j}\right)}}\times 100$$

where $n_{ij}$ is number of distinct atom pairs between the side chains of amino acid residues i and j that lie within a distance of 4.5 Å. $N_{i}$ and $N_{j}$ are the normalization factors for residues i and j, respectively.

We constructed the residue interaction networks using dynamic correlations [112] that yielded the robust network signatures of long-range couplings and communications. The ensemble of shortest paths was determined from the matrix of communication distances using the Floyd–Warshall algorithm [130]. Network graph calculations were performed using the python package NetworkX [131]. Using the constructed protein structure networks, we computed the residue-based betweenness parameter. The short path betweenness centrality of the residue i was defined to be the sum of the fraction of shortest paths between all pairs of residues that pass through the residue i:

(6) $$C_{b}(n_{i})=\sum_{j<k}^{N}\frac{g_{jk}(i)}{g_{jk}}$$

where $g_{jk}$ denotes the number of the shortest geodesics paths connecting j and *k,* and $g_{jk}(i)$ is the number of the shortest paths between residues j and *k* passing through the node $n_{i}$.

The betweenness centrality metric was also computed by evaluating the average shortest path length (ASPL) change by systematically removing individual nodes [132,133]. The following Z-score was, then, calculated:

(7) $$Z_{i}=\frac{B_{k}-\langle B\rangle }{\sigma }$$

Through the mutation-based perturbations of the protein residues, we computed the dynamic couplings of the residues and changes in the average short path length (ASPL), averaged over all modifications in a given position. The change of ASPL upon the mutational changes of each node is inspired and reminiscent to the calculation proposed to evaluate residue centralities by systematically removing nodes from the network.

(8) $$\Delta L_{i}=\langle {\left|\left|\Delta L_{i}^{node}\left(j\right)\right|\right|}^{2}\rangle$$

Where *I* is a given site; *j* is a mutation; and 〈⋯〉 denotes averaging over mutations. $\Delta L_{i}^{node}\left(j\right)$ describes the change of ASPL upon mutation j in a residue node i. $\Delta L_{i}$ is the average change of ASPL induced by all possible mutational modifications of a given residue.

The Z-score was, then, calculated for each node, as follows:

(9) $$Z_{i}=\frac{\Delta L_{i}-\langle \Delta L\rangle }{\sigma }$$

where 〈ΔL〉 is the change of the ASPL under mutational scanning averaged over all protein residues in the S-RBD and σ is the standard deviation. The ensemble-averaged Z–scores ASPL changes were computed from the network analysis of the conformational ensembles using 1000 snapshots of the simulation trajectory for the native protein system. The Girvan–Newman algorithm [134,135,136] was used to identify local communities. In this approach, edge centrality (also termed as edge betweenness) is defined as the ratio of all the shortest paths passing through a particular edge to the total number of shortest paths in the network. The method employs an iterative elimination of edges with the highest number of the shortest paths that pass through them. By eliminating edges, the network breaks down into smaller communities. The algorithm starts with one vertex, calculates edge weights for paths that pass through that vertex, and then repeats it for every vertex in the graph and sums the weights for every edge. However, in complex and dynamic protein structure networks, it is often that the number of edges can have the same highest edge betweenness. An improvement of the Girvan–Newman method was implemented, and the algorithmic details of this modified scheme were given in our recent studies [137,138]. Briefly, in this modification of the Girvan–Newman method, instead of a single highest edge betweenness removal, all highest betweenness edges are removed at each step of the protocol. This modification makes the determination of the community structure invariant to the labeling of the nodes in the graph and leads to a more stable solution. The modified algorithm proceeds through the following steps: (a) Calculate the edge betweenness for every edge in the graph; (b) Remove all edges with the highest edge betweenness within a given threshold; (c) Recalculate the edge betweenness for the remaining edges; and (d) Repeat steps b–d until the graph is empty.

## 4. Conclusions

We combined all-atom MD simulations and ensemble-based mutational scanning of the S-RBD WT complex with ACE2 as well as the S-RBD Delta and Omicron variants to identify the critical binding and allosteric hotspots as well as to dissect the mechanistic role of the mutational variant positions.

Conformational dynamics analysis revealed an increased stabilization of the binding interface for the S Omicron RBD–ACE2 complex, and an enhanced number of stable intermolecular interactions compared to the more flexible S Delta RBD complex. By employing the conformational ensembles of the S-RBD WT, Delta and Omicron variant complexes with ACE2, we performed the mutational scanning of the interfacial RBD residues and computed binding free energy changes. We found that the RBD-Delta mutational sites L452R and T478K have a minor effect on the binding affinity, while a greater contribution of the neighboring Y453, L455 and F456 residues to protein stability and RBD–ACE2 interactions may offer a moderately improved binding of the Delta variant. Our results show that N501Y is the most critical binding affinity hotspot in the S Omicron RBD complex with ACE2 and the supporting energy hotspots in this region (S496, R498 and H505) collectively form the dominant interfacial cluster with ACE2. According to our results, mutations in the S477N position produce very minor destabilizing changes, while the T478K and E484A sites contribute insignificantly to the binding interface, thereby allowing for virus flexibility to produce mutations that evade neutralizing antibodies without compromising the binding affinity with ACE2.

Through the perturbation-based scanning of the allosteric propensities of the SARS-CoV-2 S-RBD residues and dynamics-based network centrality and community analyses, we characterized the global mediating centers in the complexes and the nature of local stabilizing communities. We showed that a constellation of mutational sites (G496S, Q498R, N501Y and Y505H) correspond to key binding energy hotspots and also contribute decisively to the key interfacial communities that mediate the allosteric communications between S-RBD and ACE2. These Omicron mutations are responsible for both favorable local binding interactions and long-range allosteric interactions, providing key functional centers that mediate the high transmissibility of the virus. This study suggests that the SARS-CoV-2 S protein may exploit the plasticity of the RBD to generate escape mutants, while engaging a small group of functional hotspots to mediate efficient local binding interactions and long-range allosteric communications with ACE2.

## Figures and Tables

**Table 1:** The description of the Delta and Omicron variants examined in this study.

SARS-CoV-2 Variant	Sequence ID	Mutational Landscape	Protein Structure S-RBD Complex with ACE2
Wild-type	NCBI ID:P0DTC2		pdb id 6M0J
Delta Variant (B.1.617.2)	NCBI: QWK65230.1	T19R, G142D, Δ156-157, R158G, Δ213-214, **L452R**, **T478K**, D614G, P681R, D950N	pdb id 7WBQ, 7W9I
Omicron Variant (B.1.1.529)	GSAID ID: R40B60_BHP_3321001247/2021	A67V, Δ69-70, T95I, G142D, Δ143-145, N211I, L212V, ins213-214RE, V215P, R216E, **G339D**, **S371L**, **S373P**, **S375F**, **K417N**, **N440K**, **G446S**, **S477N**, **T478K**, **E484A**, **Q493R**, **G496S**, **Q498R**, **N501Y**, **Y505H**, T547K, D614G, H655Y, N679K, P681H, N764K, D796Y, N856K, Q954H, N969K, L981F	pdb id 7WBP, 7WBL, 7T9L

## Data Availability

Data is fully contained within the article. The data presented in this study are available in the article.
